# Protective Effects of Royal Jelly on Oxymetholone- Induced Liver Injury in Mice

**DOI:** 10.7508/ibj.2016.04.007

**Published:** 2016

**Authors:** Vahid Nejati, Ensieh Zahmatkesh, Mohammad Babaei

**Affiliations:** 1 Department of Histology and Embryology, Faculty of Science, Urmia University, Urmia, Iran; 2 Department of Veterinary Anatomy and Embryology, Faculty of Veterinary Medicine, University of Tehran, Tehran, Iran

**Keywords:** Mice, Liver, Oxidative stress, Oxymetholone

## Abstract

**Background:**

The present study was carried out to investigate the possible protective effects of royal jelly (RJ) on oxymetholone (OXM)-induced oxidative liver injuries in mice.

**Methods:**

In total, 32 adult male NMRI mice were divided into four groups of eight mice each. Mice in groups 1 and 2 were orally administered 5 mg/kg/day OXM for 30 days. At the same time, mice in group 3 received RJ at a dose of 100 mg/kg/day. Saline control and RJ control groups were also included in this study.

**Results:**

Administration of 5 mg/kg OXM resulted in a significant decrease in total antioxidant capacity and catalase activity, as well as a significant increase in malondialdehyde (*P*<0.05). In addition, OXM-administrated mice showed a slight increase in liver enzymes, including alanine amino transferase, aspartate amino transferase, and alkaline phosphatase. Although OXM caused histopathological changes in the liver, RJ could significantly improve all of the above-mentioned parameters at a dose of 100 mg/kg.

**Conclusion:**

The results of the present study indicated that RJ has a partially protective effect on OXM-induced liver toxicity in mice.

## INTRODUCTION

Liver enzymes and proteins are highly important biomarkers for the diagnosis and assessment of the normal function of tissues, organs and whole body[1]. Major or minor changes in cell membrane integrity lead to significant changes in liver enzyme activities. For example, alanine amino transferase (ALT) and aspartate amino transferase (AST) are commonly used for the diagnosis of changes in liver diseases, hepatocellular damage and increased permeability of liver cells, whereas alkaline phosphatase (ALP) is involved in extrahepatic or intrahepatic obstruction[2]. Due to the fact that the biotransformation of xenobiotic compounds is accumulated in the liver[3], it is possible to monitor the safety of xenobiotics such as trial drugs, particularly medicinal plant extracts.

Oxymetholone (OXM), a synthetic androgen analogue[4], is an appropriate medicine for patients with anemia and osteoporosis. It is also able to stimulate muscle growth in malnourished or underdeveloped individuals. Although other agents can be applied for the treatment of anemia, OXM is quite common among athletes. OXM is an alpha-17-alkylated steroid that increases the stress level in the liver. The recommended doses for muscle size and strength in a bodybuilder increase from half to one tablet one time a day for 6-12 weeks. Nevertheless, some athletes take larger doses (two or three tablets) per day. On the other hand, all anabolic androgenic steroids can result in hazardous side effects such as depression, lethargy, headache, swelling, rapid weight gain, priapism, changes in skin color, urination problems, nausea, excessive increase in glycogen reservoirs and liver cancer[5].

Royal jelly (RJ), a milky white and highly viscous fluid secreted from the salivary gland of honey bees (Apis mellifera), results from incomplete digestion of honeydew in worker bees[6] and is essential for honeybee queen development[7]. RJ contains a variety of major nutritional constituents, including proteins, sugars, free amino acids, fatty acids, minerals and vitamins[8]. In experimental animals, RJ has been demonstrated to have several physical and chemical properties, including anti-inflammatory, antioxidant, anti-tumor and immunomodulatory activities[9]. Protein fractions in RJ exhibit increased antioxidative activities and high scavenging ability against free radicals, such as superoxide anion, 1,1-diphenyl-2-picrylhydrazyl and hydroxyl radicals[10]. In a study, Inoue *et al*.[11] have indicated positive effects of RJ on oxidative deoxyribonucleic acid damage and the life span of C3H/HeJ mice. In the present study, we attempted to show the role of RJ, as a natural compound with antioxidant and immunomodulatory activities, in preventing the side effects of liver toxicity induced by OXM[8,9].

## MATERIALS AND METHODS

### Animals

In total, 32 adult male NMRI mice (8-9 weeks old, weighing 30±2 g) were obtained from the Animal House of Science Faculty, Urmia University (Urmia, Iran). All mice were allowed free access to tap water and food under controlled temperature (22±2ºC), humidity (55±5%) and normal photoperiod.

### Drugs

OXM was used at a dose of 5 mg/kg (pilot) and dissolved in saline before oral administration. At the same time, RJ was used at a dose of 100 mg/kg[12].

### Experimental design

The experimental animals were divided into four groups of eight mice each, including control, OXM, RJ and OXM+RJ. Control and OXM groups received saline (0.1 ml/mouse/day) and OXM (5 mg/kg/day) orally for 30 days, respectively. Mice in RJ group received RJ at a dose of 100 mg/kg daily, and mice in OXM+RJ group received 5 mg/kg OXM and 100 mg/kg RJ daily for 30 days.

### Sampling

Animals were sacrificed by cervical vertebrae dislocation 24 hours after the last treatment[15]. The animal studies were approved by the local Ethics Committee. Blood for biochemical analysis was drawn directly from the atrium. To obtain liver tissue samples, the abdominal cavity was opened through a vertical midline abdominal incision, and the liver was immediately excised for analysis. Livers were divided into two pieces, one of which was fixed in 10% formalin for future histological evaluation, and another was used for further biochemical studies.

### Measurement of catalase activity

Based on its capability to decompose H_2_O_2_ in liver tissue, catalase activity was measured and determined using the Aebi’s method[13]. As a rule, H_2_O_2_ decomposition can be assessed by a decrease in absorbance at 240 nm. To this end, 30 mM hydrogen peroxide and 50 mM phosphate buffer (pH 7) were used as a substrate and an alternative substrate in the blank solution, respectively. The assay solution contained 2 ml homogenate of liver tissue and 1 ml hydrogen peroxide. The reaction was initiated by the addition of H_2_O_2_, and the decrease of absorbance was evaluated by a spectrophotometer (Pharmacia, Novaspec II, and Biochrom, England) at 240 nm for 30 seconds. The values were expressed as U/g tissue.

### Measurement of malondialdehyde (MDA) levels

Lipid peroxidation was determined by the spectrophotometric thiobarbituric acid assay[14]. MDA, the end product of lipid peroxidation, reacts with thiobarbituric acid and generates a colored product, which can be measured optically at 532 nm. The results of the assay were expressed as MDA mmol/g tissue.

### Measurement of total antioxidant (ferric-reducing antioxidant power [FRAP] assay test)

The FRAP value was measured by comparing the change in absorbance at 593 nm in a test reaction mixture containing the mixture (tissue samples with 2, 4, 6-tri-(2-pyridyl)-s-triazine) with a defined ferrous ion concentration. Reactive oxygen species (ROS) resulting from a normal aerobic metabolism is potentially harmful. These free radicals are usually removed or disabled by antioxidant groups *in vivo*. When reduced to the ferrous form (FeII) in lower pH values, the FeIII-2,4,6-tri-(2-pyridyl)-s-triazine complex produces an intense blue color product with high absorption at 593 nm. In general, conditions favorable for complex development are provided in the presence of reductants (antioxidants), which allows the color development. Standard solution of ferrous sulfate (FeII100 to 2000 mM) was prepared in distilled water. Data were expressed as mmol tissue weight (FRAP value)[16,17].

### Measurement of enzymes

Serum biomarkers of liver function, including ALT, AST, ALP, albumin and total proteins[18] were measured using a spectrophotometric assays kit (Pars Azmoon Co., Tehran, Iran) and an automatic analyzer (Architect c8000 Clinical Chemistry System, USA).

### Histopathological examination

After fixing in formalin, liver tissues were embedded in paraffin, sectioned at 5-μm thickness and then stained with hematoxylin and eosin. All sections were examined by light microscopy.

### Statistical analysis

The data were presented as mean±SEM. Differences between the groups were analyzed using one-way variance analysis (ANOVA), followed by Tukey’s test using SPSS software (version 21). Values less than 0.05 (*P*<0.05) were considered to be statistically significant.

## RESULTS

### Catalase concentration

Statistical analysis results showed that in the OXM group, OXM was able to significantly decrease catalase enzyme activity, when compared to the control group (*P*<0.05). In the OXM+RJ group, RJ was able to improve this parameter in liver tissues attributed to the OXM group (Table 1).

**Table 1:** The effects of OXM and RJ on MDA, catalase activities and FRAP levels in adult male mice

Groups	MDA (mmol/g tissue)	Catalase (U/g tissue)	FRAP (mmol/g tissue)
Control	300.66±16.75^b^	21.00±1.15^b^	15.52±1.11^b^
OXM	463.33±17.48^a^	15.33±0.33^a^	9.26±0.42^a^
RJ	281.66±13.69^b^	25.33±0.88^ab^	16.48±0.88^b^
OXM+RJ	393.66±4.91^ab^	16.66±0.88^a^	12.44±0.83

a Significant differences compared to control (*P*<0.05);

b Significant differences compared to the OXM group (*P*<0.05);

OXM, oxymetholone; RJ, royal jelly; MDA, malondialdehyde; FRAP, ferric-reducing antioxidant power assay

### Malondialdehyde concentration

OXM-induced lipid peroxidation was found in liver tissue by significantly elevated MDA in the OXM group, as compared with the control group (*P*<0.05). The MDA content in the OXM +RJ group was lower than that in the OXM group (*P*<0.05) (Table 1).

### Total antioxidant concentration (ferric-reducing antioxidant power assay test)

A decrease was found in total antioxidant concentration (*P*<0.05) in the OXM group compared to the control, while OXM+RJ group showed an increase in total antioxidant concentration as compared to the OXM group (Table 1).

### Biochemical parameters in experimental animals

Table 2 represents the effect of various treatments on total proteins, albumin and liver enzymes in mice. OXM treatment alone caused a slight decrease in total proteins and albumin compared with the control group. However, mice treated with OXM+RJ showed a slight improvement in the values of these two parameters in serum compared to the OXM-treated group. In addition, OXM-treated mice indicated an increase in serum levels of ALT, ALP and AST compared to the controls, but this increase was not significant, revealing liver damage. Nonetheless, OXM+RJ-treated mice showed a slight improvement in liver damage, as compared to the mice receiving OXM alone (Table 2).

**Table 2:** The effects of OXM and RJ on ALP, ALT, AST, total protein and albomin levels in adult male mice

Groups	ALP (U/L)	ALT (U/L)	AST (U/L)	Total protein (g/dl)	Albomin (g/dl)
Control	133.33±4.91	80.93±0.93	269.53±22.48	6.48±0.09	3.80±0.26
OXM	166.00±2.08	83.36±2.88	279.53±6.18	6.14±0.14	3.33±0.19
RJ	131.33±13.53	80.30±1.90	268.00±28.71	6.51±0.10	3.89±0.15
OXM+RJ	162.33±17.36	81.93±1.03	259.96±10.42	6.21±0.06	3.97±0.07

OXM, oxymetholone; RJ, royal jelly; ALP, alkaline phosphatase; ALT, alanine amino transferase; AST, aspartate aminotransferase

### Histopathological changes in the liver tissue

**Fig. 1:**
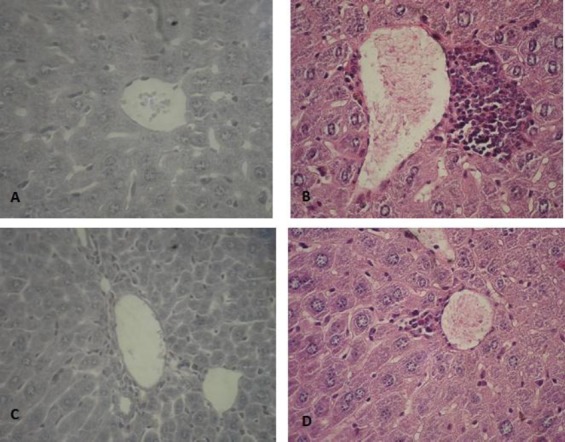
Histophatology section of liver. Cross-section of liver derived from A) a control mouse, indicating normal liver cells (H & E ×4000), B) a mouse treated with OXM (5 mg/kg), showing some parts of the inflammatory foci around the central venous (H & E ×4000), C) a mouse treated with RJ (5 mg/kg), indicating healthy liver cells (H & E ×4000), D) a mouse treated with OXM+RJ (OXM [5 mg/kg]+RJ [(5 mg/kg]), showing RJ beneficial effects on pathological changes caused by OXM (H & E ×400). H & E, hematoxylin and eosin

Some inflammatory foci were found around the central venous in the OXM group (Fig. 1B), but not in the control group (Fig. 1A). In the group that only received RJ, liver cells were healthy, and port area was normal (Fig. 1C). Additionally, the OXM+RJ group indicated that RJ is able to reduce histopathological changes (Fig. 1D).

## DISCUSSION

The use of anabolic steroids can be associated with a number of side effects[19]. Nowadays, it is clear that the abuse of anabolic-androgenic steroids can result in serious adverse effects on the liver as well as cardiovascular, central nervous, musculoskeletal, endocrine and reproductive systems[20]. The current study demonstrated that OXM leads to a significant increase in liver MDA in treated subjects as compared to the control group, highlighting the role of OXM in increased production of free radicals in liver tissue. Since lipids are the primary targets of ROS, OXM is able to induce MDA in liver tissue. MDA is the documented primary biomarker of oxidative stress and lipid damage resulting from free radicals[21]. OXM induction results in decreased levels of catalase enzyme and total antioxidant capacity in treated mice than the controls.

Hydroperoxides have toxic effects on cells either directly or through degradation into highly toxic hydroxyl radicals. They may also react with metals such as iron or copper and form stable aldehydes like MDA that damages cell membranes[22]. Therefore, it can be concluded that the negative effects of oxymetholone on oxidative stress may be due to the production of free oxygen and hydroxyl radicals by OXM.

In the present study, serum AST and ALT activities were measured to evaluate the liver injury. The result indicated that OXM increases liver enzymes AST, ALT and ALP. These liver enzymes are used as ‘markers’ to ascertain early toxic effects of administered foreign compounds on experimental animals[23,24]. ALP, as a membrane-bound enzyme, and ALT and AST, as cytosolic enzymes, are highly concentrated in the liver and kidney. In addition, they are found in significant quantities in serum when cell membrane becomes leaky or even completely ruptured[25-26]. In 2008, Rohani and Imanipoor[28] have reported that anabolic steroids can induce increased levels of ALT and AST liver enzymes.

The average daily doses and the duration of administration of testosterone and anabolic steroids taken by athletes are usually exceeded from those that are recommended for medical purposes or administered in experimental conditions. As expected, the consumption of high doses of these steroids can cause serious side effects especially for the hematological system and liver[28]. In the current investigation, histo-pathological examination revealed that OXM induces inflammation in the liver. This inflammation observed as inflammatory foci can especially be found around the central vein and can be attributed to OXM-induced free radicals in the liver[29]. Despite tremendous advances in modern medicine, prevention and treatment of liver diseases still are challenging. However, the role of oxidative stress and inflammation in the pathogenesis of hepatic diseases is well-established. Therefore, blocking or retarding the chain reactions of oxidation and inflammation can be considered as a promising strategy to prevent or treat liver injury[30].

ROS, as a common by-product of oxidative biochemical and physiological processes, is involved in numerous physiological and pathophysiological processes. Furthermore, higher concentrations of ROS can result in cell damage through oxidative modification of proteins, lipids and DNA and, therefore, plays a major role in the pathogenesis of a variety of human diseases[31]. Kim *et al*.[32] have reported that liver injury is associated with the increase of ROS generation in liver. We used RJ at a dose of 100 mg/kg, and investigated its effect on OXM-induced oxidative stress damage in liver tissue. The result indicated that RJ is able to increase significantly the levels of catalase enzyme and total antioxidant capacity and decrease the MDA levels in liver tissue. The body is equipped with a broad variety of antioxidants defense mechanisms such as superoxide dismutase, glutathione peroxidase, catalase enzymes and antioxidant vitamins (like vitamins A, C and E) that function constantly during normal metabolic and physiological processes[33]. Therefore, it can be concluded that RJ can prevent the deleterious effects of ROS due to its antioxidant properties.

RJ has the ability to decrease the levels of ALT, AST and ALP enzymes in subjects treated with RJ as compared to the OXM group, although this effect could be attributed to the RJ-contained vitamin C, vitamin E and arginine. Vitamins E and C are well-documented antioxidants that inhibit free radical-induced injury of cell membranes and decrease liver inflammation caused by OXM[33]. In 2011, Ali Karadeniz *et al*.[34] carried out an experiment on protective effects of RJ on liver and kidney of mice treated with cisplatin. They found that RJ is able to improve the adverse effects of cisplatin, including reduced liver enzymes and total antioxidant capacity.

Based on the results of this study, it can be postulated that OXM has the ability to induce histopathological changes and increase liver enzymes through increased oxidative stress in this tissue. On the other hand, RJ has the potential capacity to improve OXM-induced damages due to its antioxidant property. This protection may also be attributed to antioxidant and radical scavenging activities of RJ and its components. Therefore, RJ can be considered as a promising compound to prevent liver toxicity manifested by OXM.
